# Water Absorption Capacity and Agricultural Utility of Biopolymer-Based Hydrogels: A Systematic Review and Meta-Analysis

**DOI:** 10.1021/acspolymersau.5c00019

**Published:** 2025-06-11

**Authors:** Guilherme Schwingel Henn, Caroline Schmitz, Liliana Berté Fontana, Heloisa Vieceli Nunes Corrêa, Daniel Neutzling Lehn, Claucia Fernanda Volken de Souza

**Affiliations:** 1 Laboratory of Food Biotechnology, 186081 University of Vale do Taquari, Lajeado, ZC, RS 95914-014, Brazil; 2 Graduate Program in Biotechnology, 186081 University of Vale do Taquari, Lajeado, ZC, RS 95914-014, Brazil

**Keywords:** mass swelling, cross-linking, biopolymers, agriculture, agroindustrial waste, sustainable materials, soil conditioners, fertilizer carriers

## Abstract

This review aims to elucidate the relationship between hydrogel composition and water absorption capacity, with a focus on biobased hydrogels, the influence of their constituents on water absorption, and their relevance to agricultural applications. The most frequently used biopolymers are cellulose, starch, chitosan/chitin, and alginate, all of which are derivable from agroindustrial waste, offering sustainable and environmentally friendly sourcing. These polymers possess a high amount of hydrophilic functional groups, enhancing their affinity for water and enabling the formation of highly absorbent hydrogels. Cross-linking agents further affect the hydrogel’s swelling capacity by altering the number of available hydrophilic groups. Among them, *N*,*N*′-methylenebis­(acrylamide) is the most prevalent due to its ability to form stable networks, favoring high water absorption. However, concerns persist regarding their persistence in soil and potential environmental toxicity upon degradation. Citric acid has emerged as a promising alternative, reflecting a shift toward environmentally safer strategies. Beyond water absorption and retention, hydrogels exhibit potential as carriers for fertilizers and bioactive compounds, enabling the controlled release and availability in soil. A few studies included in this review have explored the incorporation of beneficial microorganisms, such as , , and , into hydrogel matrices, offering a clean and effective approach for agricultural enhancement that remains underexplored. This review highlights the connection between hydrogel composition and water absorption properties, identifying ecofriendly alternatives for hydrogel synthesis and applications in agriculture. It also reveals gaps in the development of sustainable, efficient hydrogels that could contribute to more environmentally friendly practices.

## Graphical abstract

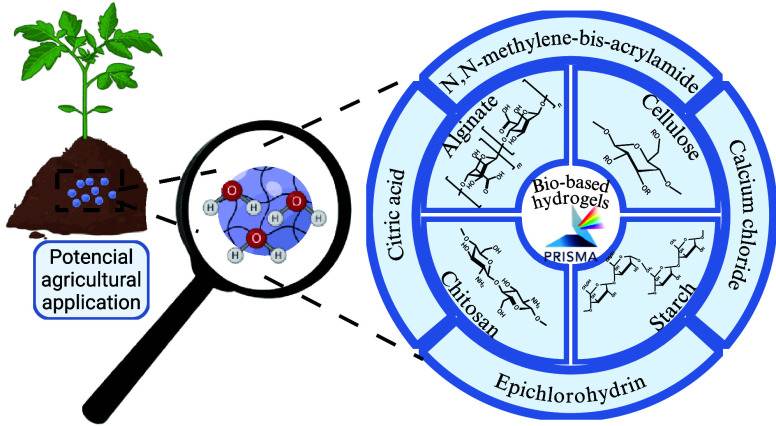

## Introduction

Hydrogels are three-dimensional, cross-linked polymer networks known for their ability to absorb and retain large amounts of water, exhibiting significant swelling capacity.^1^ Over the years, advancements in polymer cross-linking have made hydrogels highly tunable materials for a wide array of applications (Figure 1).^2,3^ In agriculture, hydrogels have emerged as promising materials to mitigate water scarcity challenges, such as drought, runoff, and irregular rainfall.

**Figure 1:**
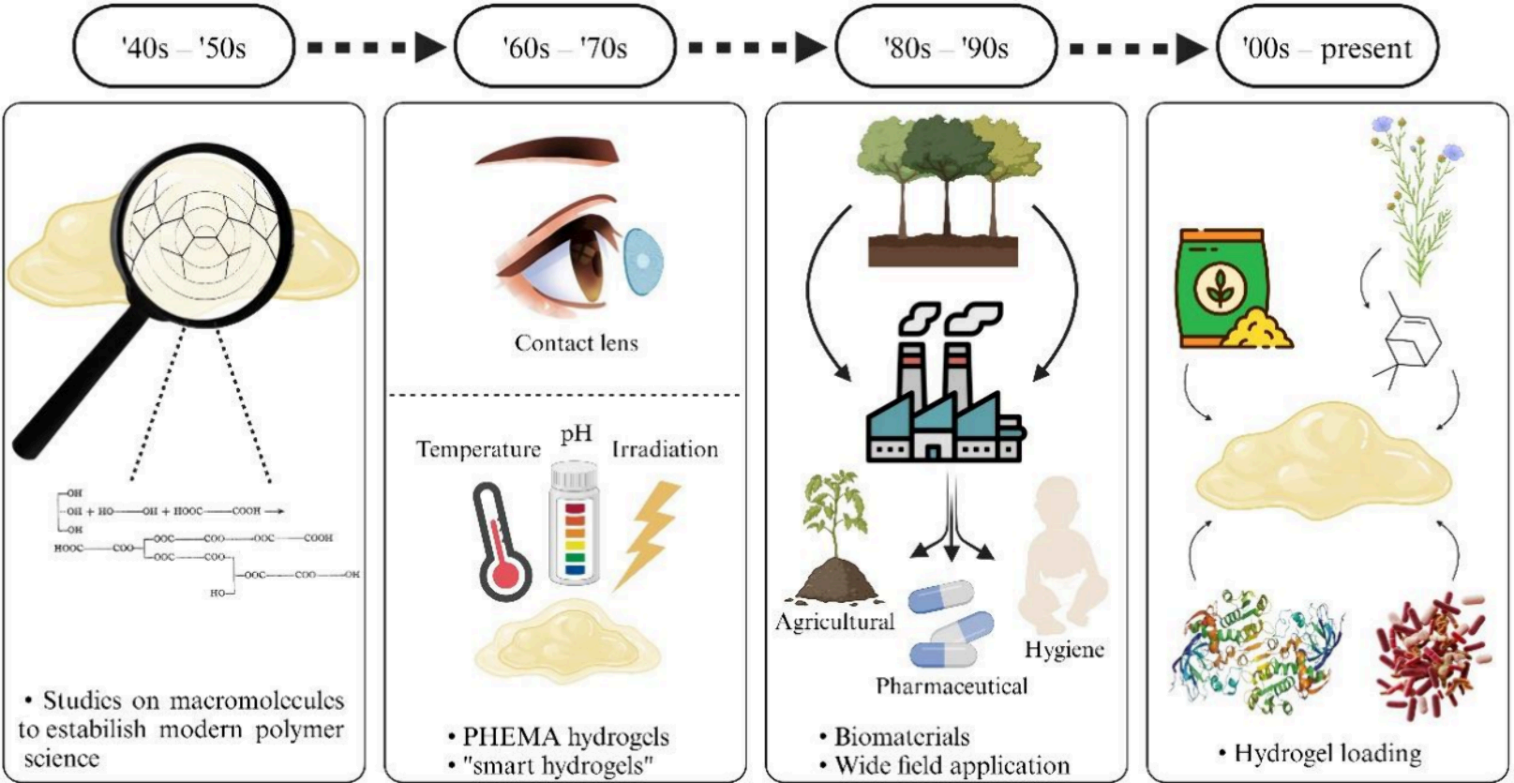
Historical development of hydrogel research and applications. This figure was created using BioRender (https://www.biorender.com/), under a Creative Commons license.

Recently, natural polymers such as polysaccharides have garnered increasing attention as sustainable alternatives to synthetic components in hydrogel production owing to their biodegradability and biocompatibility.^4,5^ These biopolymers can be sourced from a wide range of sources, including plants, animals, and microorganisms.^6,7^ Given the variability in the properties of hydrogel compositions resulting from the mixture of different polymers, there is a growing need to investigate various materials for the development of superabsorbent hydrogel formulations tailored to agricultural applications.^8^

Beyond biopolymers, cross-linking agents play an important role in determining the structure of the hydrogel network. Cross-linkers are molecules comprising two or more reactive functional groups that facilitate the formation of networks by establishing bonds between polymer chains.^9^ The hydrophilic groups of the cross-linker interact with the functional groups of the polymer, creating the interconnected network that defines the hydrogel structure.^9^ This process modifies the internal architecture of hydrogels, inducing connections across polymer chains and tuning the material’s properties.^10^

Upon exposure to water, the hydrogel network expands as water molecules infiltrate and occupy the free volume within the matrix, interacting with hydrophilic functional groups. At a state of equilibrium, the measured swelling capacity reflects the maximum volume of water that the network can absorb. Additionally, water retention capacity, measured as the rate at which water is lost under controlled temperature, relative humidity, and time, provides key insights into hydrogel performance.^11^ These water dynamics are critical to the hydrogel’s function as a soil conditioner, enhancing soil moisture retention, reducing irrigation frequency, and stabilizing plant growth under drought-prone conditions.^12–14^

Superabsorbent composites can not only serve as water reservoirs but can also function as carriers for chemicals, fertilizers, and bioactive compounds, extending their availability in the soil, protecting roots and stimulating plant development.^15–17^ Additionally, plant growth-promoting rhizobacteria (PGPR) can be immobilized within the 3D network of a hydrogel, promoting nitrogen fixation in the rhizosphere and fortifying plant defenses through the production and release of beneficial compounds.^10,18,19^

While various classifications and applications of hydrogels have been discussed elsewhere,^7,20–22^ providing insights into the different aspects and applications of these superabsorbent materials, a critical need remains for comparative evaluations that inform material selection based on specific functional requirements. To address this gap, the present work aims to systematically compile and analyze data through a meta-analysis with a focus on elucidating the relationship between structural components and water absorption capacity of hydrogels. Our findings aim to guide the development of sustainable, high-performance hydrogels for agricultural applications based on the selection of suitable components.

## Materials and Methods

### Search Strategy

This systematic review article was conducted following the Preferred Reporting Items for Systematic Review and Meta-Analysis (PRISMA) guidelines.^23^ The bibliographic search was performed in the Web of Science (WoS) and ScienceDirect databases with the following keywords: “hydrogel” OR “absorbent” OR “superabsorbent” AND “natural″ OR “organic” OR “biodegradable” AND “agriculture” OR “agronomy” OR “farm”.

The eligibility criteria for the articles were as follows: (i) Document type: only research articles were included; (ii) Language: only articles written in English were included; (iii) Publication date: only articles published between 2018 and 2024 were included. The selection process began with a review of titles and abstracts, discarding those that were not aligned with the aims of this review. The selected articles were then read in full, and articles that did not report the synthesis of hydrogels or did not present data on their mass swelling or water absorption capacities in distilled/deionized water at room temperature were excluded from the compilation.

To enable comparison between studies, water absorption capacity units were converted to g_water_/g_hydrogel_, with priority given to data from studies reporting values after approximately 24 h of immersion. ImageJ software was used to obtain the approximate value on studies where data were only available through graphs.

### Meta-Analysis

Three authors (G.S.H., L.B.F., and C.S.) conducted the meta-analysis using data extracted from the included articles. Discrepancies in the retrieved data were resolved through discussion. For studies in which more than one method was used to produce the hydrogel, each method was treated as an independent entry into the analysis. The influence of different polymers and cross-linking agents on the water absorption capacity was assessed.

All statistical analyses were performed using R (version 4.2.3, Missouri, USA)^24^, in RStudio, employing the meta package.^25,26^ A random effects model (REM) was applied to account for between-study variability.^27^ Proportion values for water absorption were summarized using the minimum and maximum values reported, and variance stabilization was achieved using the Freeman–Tukey double arcsine method. Statistical heterogeneity across studies was assessed by using the I^2^ test. For dichotomous variables, odds ratios (ORs) with 95% confidence intervals (CIs) were calculated using the Mantel–Haenszel method under an REM, regardless of the observed heterogeneity. A two-tailed *p*-value ≤ 0.05 was considered statistically significant. Random effects were estimated using the restricted maximum likelihood method, applied to predetermined variables such as biopolymer and cross-linker types. To assess potential publication bias, a funnel plot was generated and examined qualitatively. Heterogeneity was interpreted in accordance with the Cochrane Handbook for Systematic Reviews of Interventions (version 5.1.0), where I^2^ values of 25–50% were considered low, 50–75% moderate, and >75% substantial. Values above 50% were generally regarded as indicative of notable heterogeneity.^23^

## Results and Discussion

Figure 2 illustrates the research flowchart outlining the article selection process. Initially, 684 records were identified and screened across two databases: 383 from WoS and 301 from ScienceDirect. After screening, 378 records were excluded for not meeting the eligibility criteria, i.e., were non-English language articles, were not full-text papers, or were duplicated entries across databases.

**Figure 2:**
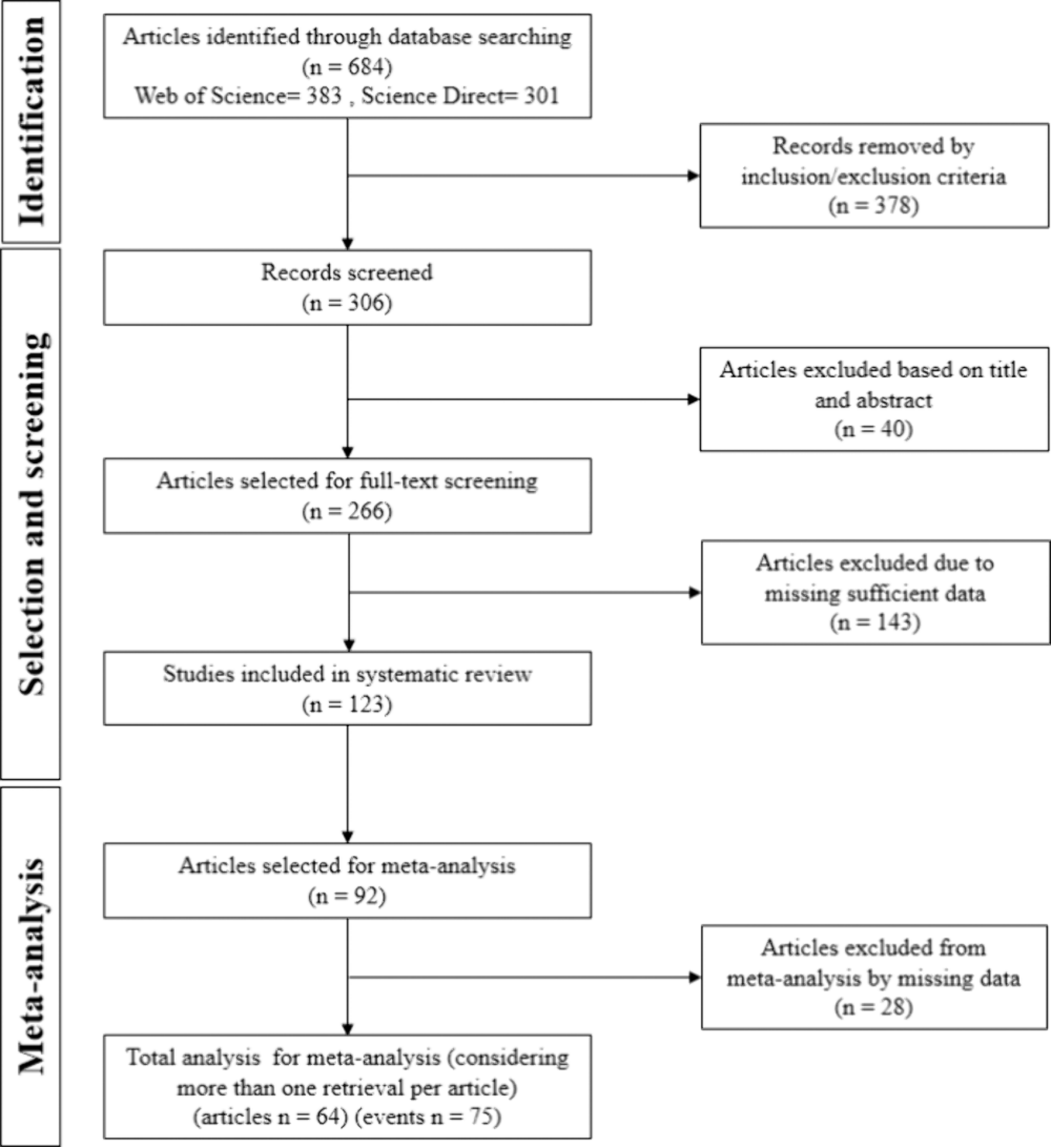
Flowchart illustrating the study selection process following the Preferred Reporting Items for Systematic Reviews and Meta-Analyses (PRISMA) guidelines. Steps of the initial screening, eligibility assessment, and final inclusion are depicted.

Out of the remaining 306 articles, 40 were excluded following the title and abstract screening, leaving 266 records for full-text examination to extract qualitative data. After this detailed examination, 143 records were further excluded due to insufficient data, i.e., the absence of natural components, lack of water absorption determination, or the mass swelling assay not being conducted using distilled/deionized water at room temperature.

Ultimately, 123 records met all of the inclusion criteria. Of these, 92 articles, which employed the most common natural polymers (cellulose, starch, chitosan/chitin, and alginate), were selected for meta-analysis. After assessment of bias and excluding articles that provided a single data point (i.e., studies that did not report both minimum and maximum water absorption values), 28 additional articles were removed. Thus, the meta-analysis was conducted on 64 articles. Some studies investigated hydrogels composed of two of the recurrent natural polymers (referred to as “events”). As a result, a total of 75 mass swelling or water absorption capacities assays were analyzed in the meta-analysis.

### Natural Polymers

The choice of polymers used in hydrogel synthesis has a significant influence on determining its water absorption properties, hydrophilicity, biodegradability, mechanical strength, and absorption/desorption capacity. While synthetic materials are often favored for creating high-performing hydrogels, environmental concerns have encouraged the use of petroleum-free, natural-based polymers.^5,28^

Figure 3 highlights the most commonly used natural materials in hydrogel production. Among these, polysaccharides are the most prevalent in hydrogel synthesis due to their abundance, renewability, and low cost. These advantageous attributes allow them to complement or replace synthetic materials in forming hydrogel matrices.^29^ Other natural materials used in the manufacture of hydrogels are listed in Table S1 (Supporting Information).

**Figure 3:**
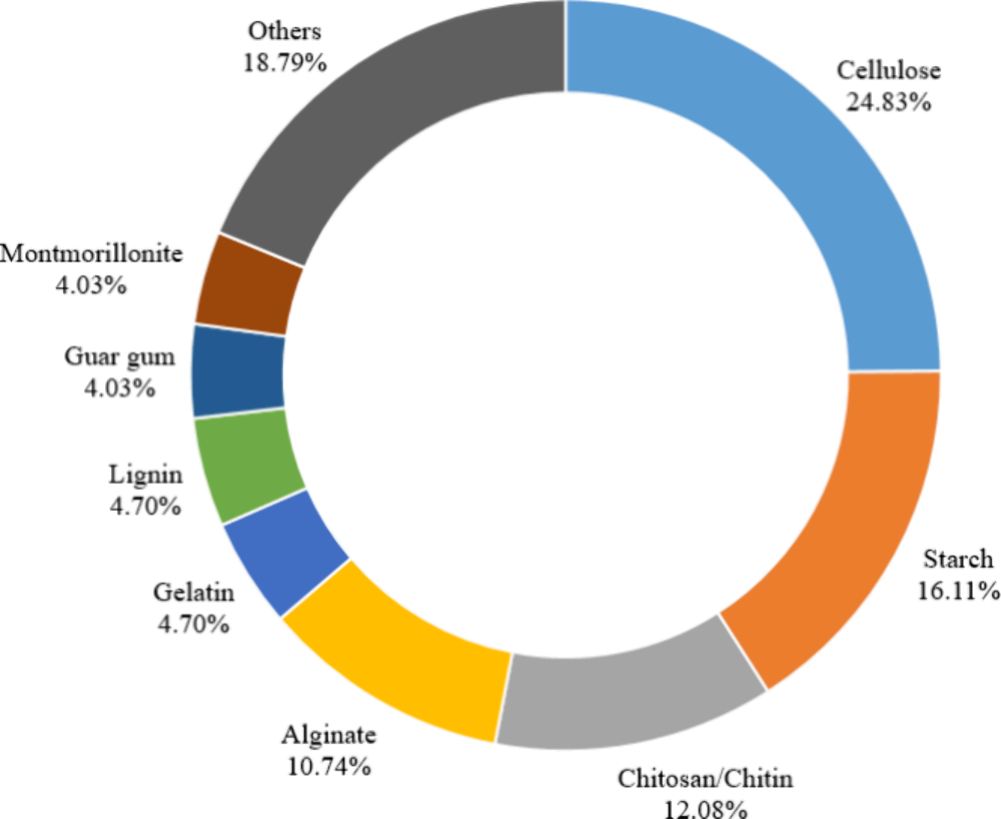
Distribution of studies utilizing the most frequently employed natural materials for hydrogel production, as identified in the Web of Science and ScienceDirect databases from 2018 to 2024, based on the Preferred Reporting Items for Systematic Review and Meta-Analyzes (PRISMA) methodology.

### Cellulose and Derivatives

Cellulose is the most commonly utilized material, featured in a total of 37 studies within this review (Table 1). In seven of these papers, cellulose serves as the sole biopolymer constituting the hydrogel, either alone or in combination with its derivatives. As a polysaccharide found in the cell walls of plants, cellulose functions as a structural component. Its abundance of hydrophilic groups enables the formation of a high number of structural bonds between polymer molecules, resulting in hydrogels with a firm and stable structure.^4,28,30^

**Table 1:** Studies with Cellulose-Based Hydrogels, Including the Adjuvant Materials and Cross-linking Agents Used, Along with the Lowest and Highest Water Absorption Capacities (WAC) and Their Respective Immersion Times in Water^a^

adjuvant materials	cross-linker	lowest WAC (g/g)	highest WAC (g/g)	immersion time (h)	references
gelatin, acrylamide, acrylic acid	MBA		150.6		^31^
gelatin, MIL-53 (Fe), acrylamide, acrylic acid		171.4*	345.8		
	epichlorohydrin		147	14.5	^32^
acid whey, polyvinyl alcohol	citric acid	3.23*	8.46*	24	^33^
coal fly ash, acrylic acid	MBA	515*	1301*	2	^34^
starch, chitosan	citric acid		4.17*	24	^35^
silk sericin, polyvinyl alcohol		22.2	26.8	24	^36^
acrylic acid	MBA	0.27	0.70	2	^37^
acrylamide	MBA		20.77*		^38^
N–S–P carbon dots, acrylamide	MBA	24.32*	25.82*		
	epichlorohydrin	7.37*	21.58*	20	^39^
soy protein isolate		1.79*	3.23*		^40^
sodium lignosulfonate, polyvinyl alcohol	ferric chloride	0.08*	0.10*	48	^41^
starch, itaconic acid, acrylic acid	MBA	219	647	12	^42^
kaolin, diatomite, acrylic acid	MBA	383*	680.45*		^43^
		101	207		^44^
poly(lactic acid)	citric acid	4.44	5.05	24	^45^
bentonite, acrylic acid, acrylamide, AMPS	MBA	750	1200		^46^
biochar, montmorillonite	citric acid	3.5*	9*	5	^47^
glycerin, dimethylacetamide	tartaric acid	39.1*	209.1*	24	^48^
keratin, EDTA, CuCl_2_		15*	41*	72	^49^
	citric acid	0.36*	3.67*	24	^50^
		48.70	78.60	48	^51^
acid whey	citric acid		14.15*	24	^52^
biochar, acrylic acid, acrylamide, AMPS	MBA	1075	1900	5	^53^
polyvinyl alcohol		5*	8.23*	4	^54^
gelatin, acrylic acid, acrylamide	MBA	39	825	24	^55^
acrylic acid	MBA	255*	342	4	^56^
humic acid, clay		19*	189*	24	^57^
alginate, MIL-100 (Fe)	calcium chloride	27.3*	54.4*	5	^58^
zein	citric acid	0.278*	0.309*	3	^59^
acid whey	citric acid	8.27*	23.87*	24	^60^
lignin, polyvinyl alcohol	epichlorohydrin	180*	1220	72	^61^
paraffin, β-cyclodextrin	epichlorohydrin	-	8.725	3	^62^
acrylic acid	MBA	33	165	-	^63^
	citric acid	45.44	200	24	^64^
chitin, montmorillonite, acrylamide	MBA	0.12	7.87	24	^65^
			12.82	6	^66^
acrylamide	MBA		14.57	6	
acrylamide	MBA	7.97*	11.02*	24	^67^

a Values with (*) represent data collected with ImageJ. WAC: water absorption capacity; AMPS: (2-acrylamido-2-methylpropanesulfonic acid); EDTA: ethylenediaminetetraacetic acid; MBA: *N*,*N*′-methylenebis­(acrylamide); PEGDE: poly­(ethylene glycol) diglycidyl ether.

The structure of cellulose can be enhanced through chemical modification to form cellulose derivatives by altering the availability of hydroxyl groups (Figure S1). The number of hydroxyl (−OH) groups in the cellulose-repeating unit facilitates reactions with cross-linking agents, ultimately determining the structure and properties of the hydrogel. The most commonly used derivatives include sodium carboxymethylcellulose (CMC) (*n* = 19), hydroxyethylcellulose (HEC) (*n* = 8), methyl 2-hydroxyethyl cellulose (*n* = 1), and ethylcellulose (*n* = 1).

Das et al.^50^ produced a hydrogel using a 3:1 ratio of carboxymethylcellulose and hydroxyethylcellulose (CMC:HEC) combined with cellulose nanocrystals. The authors found that incorporating cellulose nanocrystals improved the water absorption capacity of the hydrogels, particularly at a concentration of 3% nanocrystals and 2% citric acid as a cross-linker, achieving a mass swelling of over 3 g/g in 24 h. This enhancement occurs because cellulose nanocrystals act as reinforcing fillers within the hydrogel network with their abundant surface hydroxyl groups contributing to improved swelling properties.

Similarly, Djafari Petroudy et al.^64^ used cellulose nanofibers extracted from wheat straw in combination with carboxymethylcellulose and hydroxyethylcellulose. They observed that increasing the carboxymethylcellulose content led to an enhanced water absorption capacity, reaching up to 200 g/g. This improvement is attributed to the high hydroxyl content of carboxymethylcellulose, which, at higher concentrations, allows more water molecules to interact with the hydrogel matrix.

Cellulose is an abundant and renewable polymer that can be sourced from a wide variety of biomass and waste sources, significantly reducing fabrication costs. Agricultural residues such as corn stalk pith,^48^ hyacinth fibers,^63^ sugar cane bagasse,^65^ and wheat straw,^64^ along with industrial byproducts such as wastepaper,^42^ offer economically and environmentally sustainable routes for cellulose extraction. The use of such agroindustrial waste not only reduces hydrogel production costs but also aligns with green chemistry principles and sustainable material development goals.^56,68,69^

For instance, E et al.^32^ prepared cellulose nanofibers from waste wood scraps and combined them with carboxymethylcellulose to create a hydrogel designed for urea loading. This hydrogel demonstrated a mass swelling capacity of 147 g/g over 870 min and retained water for up to 16 days. Durpekova et al.^52^ employed acid whey, a byproduct of cheese production, as a dispersive medium to prepare a cellulose-based hydrogel, which demonstrated a swelling capacity of 14.15 g/g and retained soil moisture for over 20 days. In a follow-up study, the inclusion of 20% (w/w) poly­(lactic acid) to the formulation reduced the water absorption capacity to 4.44 g/g but notably improved water retention, sustaining 28% moisture at 28 days. While poly­(lactic acid) enhanced the structural rigidity of the hydrogel network, its lack of hydrophilic moieties limited water diffusion within the matrix.^45^

Diaz-Ramirez et al.^51^ employed a biopolymer produced by . The authors investigated the bacterial growth in static and agitated cultivation conditions, resulting in the production of hydrogels with water absorption capacities of 48.7 and 78.6 g/g, respectively. This sphere-like cellulose features a layered and porous structure with a high surface area, favoring its mass swelling capacity. These characteristics extend its potential application as a fertilizer carrier.^51,70^

Among the cellulose-based hydrogels included in this review, the combination of cellulose and poly­(vinyl alcohol) (PVA) has often been used as copolymers for the synthesis of hydrogels (*n* = 5). PVA, a nontoxic and biodegradable synthetic material, is employed to improve the structural integrity, swelling properties, and durability of hydrogels. These enhanced characteristics ensure that the hydrogel maintains a stable structure capable of swelling in water without dissolving.^71,72^

The molecular weight of substances used in hydrogel production significantly influences their absorption capacity. Huang et al.^61^ investigated the effects of varying molecular weights of hydroxyethylcellulose, PVA, and lignin on the water absorption capacity of hydrogels. After 72 h, hydrogels exhibited a mass swelling capacity of 1200 g/g. The optimal results were achieved using hydroxyethylcellulose with a molecular weight of 720,000 g/mol and PVA with 205,000 g/mol. While a high molecular weight for hydroxyethylcellulose and a low molecular weight for PVA enhanced cross-linking connections, this increased the yield but reduced the overall swelling capacity.

Similarly, Kassem et al.^54^ developed a hydrogel blend of cellulose nanocrystals and PVA for coating nitrogen, phosphorus, and potassium (NPK) fertilizers, achieving a mass swelling capacity of 8.23 g/g within 4 h and maintaining soil moisture for over 20 days. Additionally, the NPK fertilizer coated with cellulose nanocrystals and the PVA hydrogel showed a significant increase in crushing strength, up to 70 N, compared to the uncoated fertilizer (50 N). Fabian et al.^33^ reported similar results in a hydrogel made from carboxymethylcellulose (CMC) and PVA using acid whey as a dispersive medium. The presence of PVA improved the strength of the absorbent, yielding a mass swelling of 8.46 g/g.

Cellulose is a highly abundant biopolymer, obtained through either plant extraction or bacterial synthesis, with its structure varying depending on the source. Cellulose-based hydrogels are environmentally friendly and biodegradable, allowing safe application. The water absorption capacity of the formed hydrogel depends on the judicious selection of copolymers and cross-linking agents, altering substituent groups, blending components, and availability of hydrophilic sites. Additional properties, such as swelling capacity and structural strength, can also be adjusted by selecting the appropriate copolymers and cross-linking agents. These tunable traits make cellulose a frequent choice for the development of biobased hydrogels.^72,73^

### Starch

Starch, the second-most utilized natural polymer, was featured in 24 studies (Table 2), blended with either monomers (*n* = 1) or biomaterials (*n* = 22). Starch is a carbohydrate produced by plants as an energy reserve. Its structure consists of d-glucose units, amylopectin, and amylose, connected by glycosidic linkages (Figure S2), and contains a high number of hydroxyl groups that favor hydrogel networking.^3^

**Table 2:** Studies with Starch-Based Hydrogels, Adjuvant Materials, and Cross-Linking Agents Used and the Results of the Lowest and Highest Water Absorption Capacities (WACs) with Respective Immersion Times in Water^a^

adjuvant materials	cross-linker	lowest WAC (g/g)	highest WAC (g/g)	immersion time (h)	references
chitosan	citric acid		3.87*	24	^35^
chitosan, wood ash			3.28*	24	
chitosan, cellulose			4.17*	24	
cellulose, itaconic acid, acrylic acid	MBA	219	647	12	^42^
	citric acid		1.25	24	^74^
poly(methyl methacrylate)	trimetaphosphate	0.68*	8.43*		^75^
alginate	calcium chloride	7.02*	8.23*	20	^76^
collagen, phenylalanine, Zn(NO_3_)_2_	glycerol ethoxylate, hexamethylenediisocyanate		37.5		^77^
collagen, histidine, Zn(NO_3_)_2_			49		
collagen, threonine, Zn(NO_3_)_2_			33		
	citric acid	3	5	24	^78^
gellan gum		8*	66.5*	24	
gellan gum, bentonite		5*	55.5*	24	
gellan gum, halloysite		4*	4.5*	24	
bentonite		3*	4.5*	24	
halloysite		3.5*	4*	24	
biochar, acrylic acid	epichlorohydrin	28.18*	244.47		^79^
natural rubber, montmorillonite, acrylamide	glutaraldehyde	46.57	70.74	1	^80^
agar, gelatin, AMPS	MBA	13.5*	91.4*	24	^81^
biochar, acrylic acid	MBA		155	12	^82^
itaconic acid, acrylic acid	MBA	154	650		^83^
acrylic acid, acrylamide	MBA		440*	72	^84^
alginate, acrylamide	MBA	7.5*	15.3*	24	^85^
alginate, bentonite, AMPS, acrylic acid	MBA	610*	1484	4	^86^
bentonite, acrylamide	MBA	59.1*	94.2	4	^87^
natural rubber, acrylic acid, polyvinyl alcohol		2.20	6.76	0.67	^88^
alginate, acrylamide	MBA		201	24	^89^
chitosan	citric acid	1.01*	1.54*	24	^90^
natural char, acrylic acid, acrylamide	MBA	66.2	334.3	1	^91^
sodium borate, polyvinyl alcohol		2.3*	10.4*	24	^92^
natural rubber, polyvinyl alcohol, butanediol		1.20	2.50	7	^93^
biochar, acrylic acid	MBA	0.72*	8.08		^94^
montmorillonite, acrylic acid	MBA	3.8*	26.4*	7	^95^

a Values with (*) represent data collected with ImageJ. WAC: water absorption capacity; AMPS: (2-acrylamido-2-methylpropanesulfonic acid); MBA: *N*,*N*′-methylenebis­(acrylamide).

Ilyasov et al.^84^ synthesized a hydrogel by grafting acrylic acid and acrylamide monomers onto the starch backbone. The resulting absorbent exhibited a mass swelling capacity of more than 400 g/g. However, the properties of starch-based hydrogels can be influenced by other components, as demonstrated by Bora and Karak.^83^ In their study, itaconic acid was used with starch, and it was observed that as the itaconic acid content increased, both grafting cross-linking and swelling capacity were reduced. This reduction occurred because higher itaconic acid content increased the hydrophobicity of the hydrogel due to the presence of un-ionized carboxylic acid groups in the polymer chain, leading to lower mass swelling values.

Xiong et al.^86^ employed hydroxyethyl starch as the polymeric backbone, combined with bentonite and 2-acrylamido-2-methylpropanesulfonic acid (AMPS), to produce a hydrogel. The authors observed that a concentration of 0.14% w/w of hydroxyethyl starch achieved a peak mass swelling capacity of 1483 g/g after 4 h of immersion. Deviations in concentration, whether lower or higher, negatively impact the hydrogel network, diminishing its absorption capacity. Furthermore, an increase in AMPS concentration up to 0.3% w/w enhanced the swelling capacity due to the higher number of hydrophilic groups. A similar trend was observed by Singh et al.:^81^ as the AMPS content increased from 0.386 to 0.772 mol/L in a starch and gelatin hydrogel, the mass swelling capacities ranged from 14.28 to 133.6 g/g. The sulfonic acid groups (−SO_3_) introduced by AMPS are known for their high affinity for water, making them potent enhancers of a material’s water absorption capacity.

Modifications to the hydrogel structure can further enhance its properties. For instance, Gou et al.^79^ employed sulfonated starch to develop a hydrogel with a mass swelling capacity of up to 244.47 g/g. Their study found that varying the molar ratio of chlorosulfonic acid to the glucose units of starch influenced the structural density of the hydrogel. The degree of sulfonation affects the hydrogel’s capacity to absorb water: a lower degree of sulfonation limits the hydrogel’s interaction with water, creating a more compact network, while a higher degree of sulfonation forms larger pores, hindering its capacity to retain water molecules. The increased surface area improves water absorption by allowing more water molecules to interact with the structure.

Several studies have explored blends that enhance the porosity and density of the hydrogel structure using additives such as bentonite (*n* = 4), biochar (*n* = 3), montmorillonite (*n* = 2), and sodium borate (*n* = 1). Qin et al.^92^ investigated the properties of a hydrogel composed of starch, PVA, and borax. They observed that the inclusion of borax increased the swelling ratio by altering the crystallinity of the starch and PVA networks, leading to the formation of more amorphous regions in the hydrogel structure.

Yang et al.^105^ investigated the incorporation of biochar into starch-based hydrogels, creating an absorbent material with a highly porous structure. The increased porosity led to a larger surface area within the hydrogel network, enhancing the mass swelling capacity to 155 g/g. When applied to sandy soil at a concentration of 1% w/w, the hydrogel extended water retention for over three months and enhanced the formation of soil aggregates. Similarly, Salimi et al.^91^ used chemical oxidation on natural char to produce nanoparticles with uniform size and superior surface properties. These nanoparticles were blended with starch to create a porous composite with a mass swelling capacity of up to 334.3 g/g, capable of retaining water for up to 7 days.

Jumpapaeng et al.^80^ explored a hydrogel blend of montmorillonite (MMT), starch, and natural rubber. Initial increases in MMT content improved the mass swelling capacity from 56.90 to 70.74 g/g, attributed to repulsive forces between negatively charged MMT surfaces and −CONH_2_ groups in the hydrogel network, creating a more expansive gel structure and improving the water absorbency. However, further additions of montmorillonite reduced water absorption, as the MMT began to act as a cross-linker, increasing the network density. Zhou et al.^95^ reported comparable behavior in a starch-MMT hydrogel, achieving a mass swelling capacity of 102.6 g/g capable of retaining water in soil for 12 days.

Overall, starch is an environmentally friendly, highly available, and biodegradable compound, making it a strong candidate for hydrogel formulations. Its ability to blend with various materials enables the enhancement of both swelling capacity and structural characteristics for the development of an efficient absorbent material.^2^ Food waste streams can serve as valuable sources of starch, offering pathways toward cost-effective production of hydrogels and contributing to the development of sustainable and environment-friendly agriculture.^95^

### Chitosan/Chitin

Chitosan and chitin were used in 19 studies included in this review (Table 3). Chitin is the second most abundant naturally occurring polysaccharide, found in the cell walls of fungi and the exoskeleton of arthropods. Chitosan is produced through the deacetylation of chitin via an alkalization process at high temperatures.^6,96^

**Table 3:** Studies with Chitosan and Chitin-Based Hydrogels, Adjuvant Materials, and Cross-Linking Agents and the Results of the Lowest and Highest Water Absorption Capacities (WACs) with Respective Immersion Times in Water^a^

adjuvant materials	cross-linker	lowest WAC (g/g)	highest WAC (g/g)	immersion time (h)	references
starch	citric acid		3.87*	24	^35^
starch, wood ash	citric acid		3.28*	24	
starch, cellulose	citric acid		4.17*	24	
cellulose, montmorillonite, acrylic acid	MBA	0.12	7.87	24	^65^
alginate	calcium chloride	3.73	6.90*	5	^82^
starch	citric acid	1.01*	1.54*	24	^90^
sodium lignosulfonate	epichlorohydrin, calcium chloride		62	24	^97^
			0.38	24	^98^
alginate, acrylamide	potassium persulfate		0.93	24	
Pluronic F-127			0.81	24	
benzaldehyde			24.57*		^99^
benzaldehyde, urea			21.57*		
benzaldehyde, guava leaves extract, cerium nitrate hexahydrate			11.10*		
montmorillonite, polyvinyl alcohol	glutaraldehyde	32.56*	83.64*	24	^100^
K-carrageenan	aluminum chloride, potassium chloride	0.27*	0.29*	14	^101^
poly(2-ethyl-2-oxazoline)	2-hydroxyethyl methacrylate	2.82*	6.03*		^102^
OSSC, green tea, acrylic acid	MBA	222*	410	4	^103^
OSSC, acrylic acid	MBA	175.75	510.35	4	^104^
biochar, acrylic acid	MBA		172	12	^105^
OSSC, polydimethylurea phosphate, acrylic acid	MBA		412*	4	^106^
aluminum chloride	formaldehyde	0.63*	1.71*	41	^107^
silica nanoparticles, acrylic acid	APTES	0.22*	18.15	4	^108^
sodium polyacrylate	epichlorohydrin	46.2*	404.3*	-	^109^
gelatin, polyvinyl alcohol			10.8	24	^110^

a Values with (*) represent data collected with ImageJ. WAC: water absorption capacity; APTES: 3-aminopropyltriethoxysilane; MBA: *N*,*N*′-methylenebis­(acrylamide); OSSC: oil shale semicoke.

Abdeen et al.^102^ synthesized a hydrogel using poly­(2-ethyl-2-oxazoline) on a chitosan backbone and evaluated its water absorption behavior with varying chitosan contents. The authors observed that as the chitosan content increased from 1% to 10% (w/w), the mass swelling capacity decreased from 6.03 to 2.82 g/g. This reduction was attributed to the increased network density formed by hydrogen bonds between chitosan OH and NH_2_ groups, which limited water diffusion within the hydrogel matrix.^108^ Additionally, He et al.^109^ reported that pure chitosan hydrogel exhibited a lower mass swelling capacity (46.2 g/g) compared to formulations incorporating sodium polyacrylate (152.1–404.3 g/g). The enhanced water absorption in the latter was due to the abundant COO^–^ groups in sodium polyacrylate, which could hold more water molecules than the NH_2_ groups of chitosan. Conversely, Peidayesh et al.^90^ observed that increasing the chitosan content in a chitosan-starch hydrogel enhanced water absorption, reaching a mass swelling capacity close to that of pure chitosan hydrogel (1.54 g/g). This behavior is attributed to the amorphous structure of chitosan, which increases the free volume and facilitates the diffusion of water molecules.

The addition of oil shale semicoke (OSSC) has also been shown to enhance water absorption. Studies by Liu et al.^104,106^ demonstrated that incorporating 10% w/w OSSC into chitosan-based hydrogels, as well as 15% w/w OSSC,^103^ resulted in significantly high water absorption capacities, exceeding 400 g/g. OSSC enhances the hydrogel’s three-dimensional network, reducing the loss of swelling capacity after multiple swelling/drying cycles.^106^

The degree of chitosan deacetylation, which influences its molecular weight, swelling properties, and mechanical strength, determines its performance.^111^ Owing to its biodegradability, biocompatibility, and nontoxicity, chitosan is a promising natural polymer for applications in agriculture.^96,112^

### Alginate

Alginate was used in 18 studies (Table 4), with three of them focusing on single alginate-based hydrogels and 15 on blends with monomers and/or biocomponents. In nature, alginate is a polysaccharide commonly found in the form of sodium salts in the cell walls and intercellular mucilage of various species of brown algae as well as in some bacteria such as *Pseudomonas*.^113^ Structurally, alginate consists of linear homopolymeric and heteropolymeric blocks of (1,4)-linked β-D-mannuronate (M) and α-L-guluronate (G) residues. The M and G residues of alginate can link together in different arrangements, such as −GG–, −MM–, or −MG– blocks. Additionally, the source of the alginate influences the length of these structures and their linking pattern.^114–118^

**Table 4:** Studies with Alginate-Based Hydrogels, Adjuvant Materials, and Cross-Linking Agents Used and the Results of the Lowest and Highest Water Absorption Capacities (WACs) with Respective Immersion Times in Water^a^

adjuvant materials	cross-linker	lowest WAC (g/g)	highest WAC (g/g)	immersion time (h)	references
cellulose, MIL-100 (Fe)	calcium chloride	27.3*	54.4*	5	^58^
	calcium chloride		6.50*	20	^76^
starch	calcium chloride	7.02*	8.23*	20	
biochar, acrylic acid	MBA		188	12	^84^
acrylamide, starch	MBA	7.5*	15.3*	24	^85^
starch, bentonite, AMPS, acrylic acid	MBA	610*	1484	4	^86^
chitosan	epichlorohydrin, calcium chloride		62	24	^97^
chitosan, acrylamide	potassium persulfate		0.93	24	^98^
chitosan	calcium chloride	3.73	6.90*	5	^82^
acrylamide	MBA, calcium chloride	23.01	66.26		^116^
γ-polyglutamic acid	polyglycol diglycerol	60.34	364.12	24	^117^
biochar	calcium chloride	0.77	1.41		^118^
UIO-66, polydopamine	calcium chloride		1.61*	72	^119^
	urea, EDC, NHS	522*	729*	14	^120^
acrylic acid, acrylamide	TMPT	0.155	0.529	24	^121^
biochar, polyvinyl alcohol	calcium chloride	0.493*	0.687*	96	^122^
	calcium chloride		46.09		^123^
acrylamide	MBA	25.8*	109.3	24	^124^
natural soil colloid, acrylamide	MBA, calcium sulfate	34.1*	42.8*	24	^125^
acrylamide	MBA, calcium sulfate		47.7*	24	

a Values with (*) represent data collected with ImageJ. WAC: water absorption capacity; AMPS: 2-acrylamido-2-methylpropanesulfonic acid; EDC: *N*-(3-(dimethylamino)­propyl)-*N*′-ethylcarbodiimide hydrochloride; MBA: *N*,*N*′-methylenebis­(acrylamide); NHS: *N*-hydroxysuccinimide; TMPT: trimethylolpropane trimethacrylate.

In several studies, alginate-based hydrogels were used to load various chemicals. For example, Niu et al.^120^ used urea as a fertilizer load in alginate hydrogels. The study revealed that increasing urea content led to a mass swelling capacity of up to 700 g/g, which was attributed to the enhanced hydrogel porosity, allowing for better water diffusion. Joshi et al.^123^ produced pure alginate hydrogel beads with a mass swelling capacity of 46.09 g/g. When these beads were loaded with KNO_3_, K_2_HPO_4_, fish emulsion, and , the hydrogels could absorb 33.50 26.78, 11.80, and 42.00 g/g, respectively. These findings suggest that the high number of hydrophilic groups in alginate facilitates easy modifications for entrapping and controlling the release of compounds while maintaining significant water absorption capacity.^114,126^

The combination of alginate with biochar derived from pyrolyzed cotton straw has been shown to enhance the structural integrity of the material.^82^ The phenomenon in question arises from the porous nature of biochar, which serves to enhance the hydrogel structure and surface area, facilitating water diffusion. Consequently, the alginate-biochar hydrogel demonstrated a water absorption capacity of up to 188 g/g, with a water retention capacity exceeding three months. However, Uysal et al.^122^ observed that increasing biochar content in an alginate-biochar blend only slightly improved the mass swelling capacity. In this study, biochar was produced by pyrolysis of vine pruning waste, dry compost, and redbud leaves, resulting in highly ordered cubic structures that influenced the hydrogel’s absorption capacity.

Wang et al.^58^ investigated an alginate-cellulose hydrogel incorporating various amounts of MIL-100 (Fe) as a metal–organic framework. The study revealed that as the MIL-100 (Fe) content increased to 10%, the hydrogel’s mass swelling capacity improved to 54.4 g/g. The high amorphous and porous morphology of the hydrogel, attributed to the incorporation of MIL-100 (Fe), contributed to a larger surface area, thus enhancing the diffusion of water through the matrix.

Alginate-based hydrogels possess excellent properties, such as biocompatibility, biodegradability, and tunability, making them highly desirable for a wide range of applications. Depending on specific requirements, alginate-based hydrogels can be adapted and optimized by incorporating monomers or various compounds to enhance their structure and performance.^115,126^

### Cross-Linkers

Cross-linking is a process that induces covalent bonds between polymer chains, resulting in a more robust and durable material. These bonds increase the material’s resistance to temperature fluctuations, enhancing its thermal stability and ensuring the expected mechanical properties required of the hydrogel. Cross-linking can be initiated in three different ways, depending on the reaction trigger mechanism: thermal, physical (γ radiation, UV light), or chemical (through the addition of cross-linking agents).^10,127^ Among the most commonly used cross-linking agents are *N*,*N*′-methylenebis­(acrylamide) (MBA) (*n* = 50), citric acid (*n* = 14), epichlorohydrin (*n* = 9), and calcium chloride (CaCl_2_) (*n* = 9).

Following the meta-analysis assessment, a total of 64 articles were selected for quantitative evaluation, encompassing 75 events centered on the water absorption capacities of hydrogels produced from various biomaterials and cross-linking agents, ensuring diversity and robustness across experimental conditions. Assessment via Funnel plot analysis supports the absence of publication bias (Figure S3). According to the meta-analysis of all events, the I^2^ value for water absorption capacity among the different biopolymers and cross-linkers was 0%, indicating minimal variation between the samples when considered collectively (Figure S4).

The general figure of the meta-analysis demonstrates that regardless of the biopolymer used all exhibited a positive impact on water absorption (Figure S4). However, the observed OR values vary between different polymers and even between samples of the same polymer. This variation is attributed to the fact that, in addition to the four biopolymers reviewed here, 15 cross-linkers were also used in hydrogel formulations, which directly affects water absorption. As a result, we decided to analyze each biopolymer separately to assess its impact on water absorption when combined with various cross-linkers.

A total of 32 events were identified for cellulose, 21 events for starch, 11 events for chitosan and chitin, and 11 events for alginate. The four polygons (one for each biopolymer) show varying sizes with larger polygons contributing more to the overall summary. Among the four biopolymers, chitosan/chitin demonstrated the lowest water absorption capacity, ranging from 0 to 510 g/g. For alginate, the water absorption capacity ranged from 0 to 1483 g/g, with only the cross-linker MBA yielding the highest water absorption capacity.^86,124^ Given that chitosan/chitin and alginate had fewer events across different cross-linkers and lower water absorption capacities, only the results for cellulose and starch will be presented in the meta-analysis.

For cellulose, seven different cross-linkers were reported: citric acid (CA) (7 events), MBA (13 events), epichlorohydrin (ECH) (2 events), and no cross-linkers (7 events). Additionally, ferric chloride (FeCl_3_), tartaric acid (TA), calcium chloride (CaCl_2_), and ECH_ absorbency were each reported in a single event (Figure 4). The forest plots revealed variations in water absorption among cellulose-based hydrogels across these cross-linkers, with ECH showing the highest effect on water absorption, followed by MBA, citric acid, and no cross-linker [ORs 4.37, 2.39, 2.43, and 2.22, respectively].

**Figure 4:**
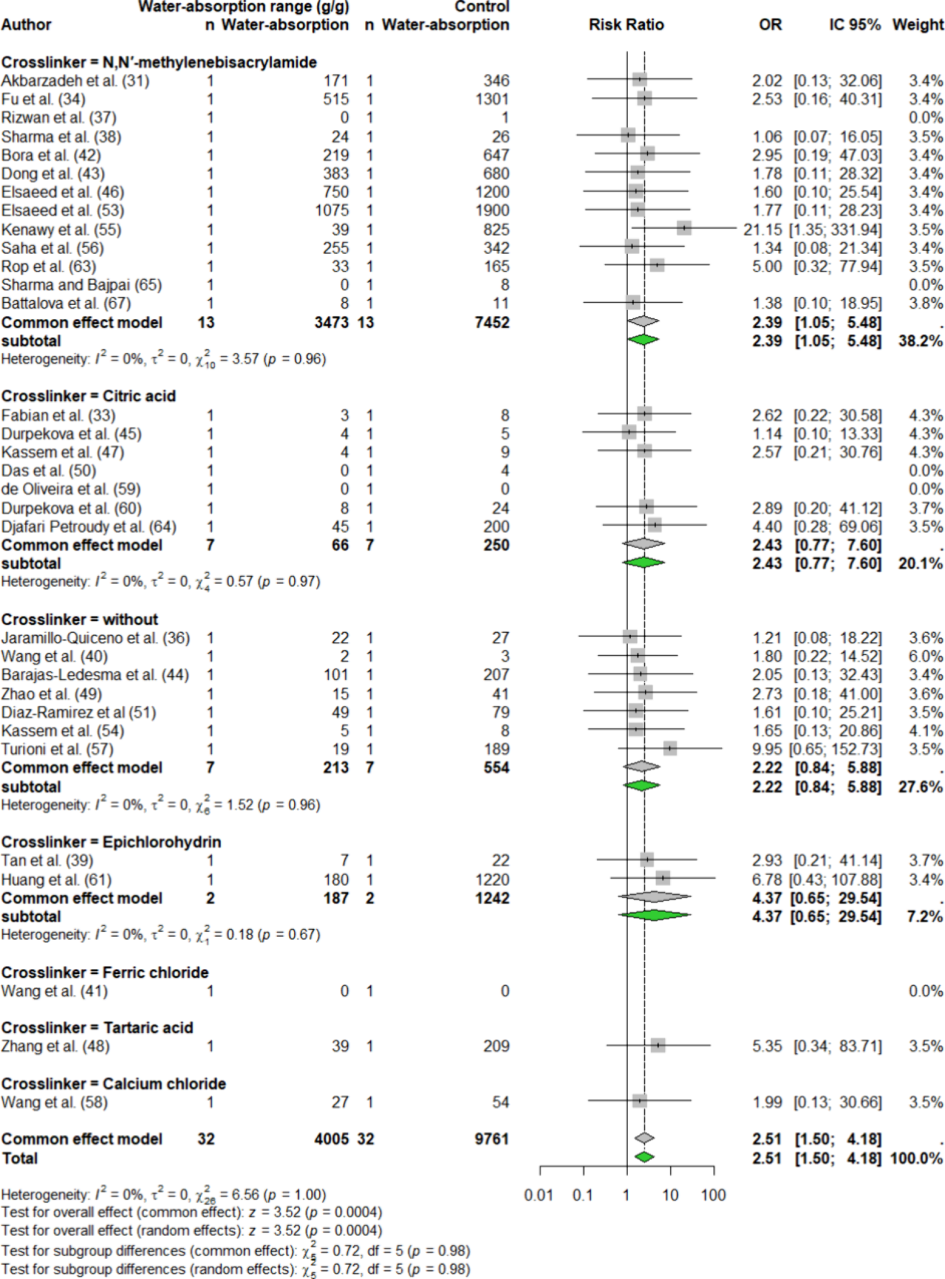
Forest plot representing cellulose-based hydrogels, detailing maximum and minimum water absorption capacities (g/g), odds ratios (ORs), summary effects, 95% confidence intervals (CIs), and the percentage of weight attributed to each study. The analysis follows an REM with a 95% CI for the mean effect size under fixed-effect meta-analysis.

A common trend observed in several studies is that low amounts of cross-linking agents lead to a weak superabsorbent network, causing the hydrophilic polymer chains to dissolve in an aqueous medium. This inefficient network formation results in fewer cross-link points, reducing the polymer’s binding affinity for water and consequently lowering its mass swelling capacity. Conversely, an excess of cross-linking agents causes a high chain density, which limits network expansion and reduces the gaps in the three-dimensional network, ultimately decreasing the water retention capacity of the hydrogels.^53,128^

The minimum water absorption recorded was 1 g/g, and the maximum reached 1900 g/g (Figure 4). The overall effect is significant (*p* = 0.0005), confirming the influence of the biopolymer and cross-linker combination on the water absorption capacity. Of the 13 events reported for MBA in combination with cellulose, eight exhibited water absorption capacities of up to 200 g/g, which represent the highest values observed across the other two subgroups (citric acid and no cross-linker).

In addition to its effectiveness in forming a robust hydrogel structure, MBA requires a lower quantity to establish bonds in the network compared to other cross-linking agents, making it a promising option for producing cellulose-based hydrogels with a high water absorption capacity. Citric acid, the most widely used naturally occurring cross-linking agent, is a nontoxic and cost-effective hydroxycarboxylic acid primarily found in citrus fruits. It forms ester bonds between polymer chains through its amino and hydroxyl groups, enhancing both the mass swelling and mechanical properties of the hydrogel network.^129^

Hydrogels formulated with cellulose and citric acid tend to show a reduced water absorption capacity. Djafari Petroudy et al.^64^ reported a significant increase in water absorption capacity, from 45 g/g to 200 g/g, upon the rise of carboxymethylcellulose content. In contrast, Turioni et al.^57^ achieved a similar increase in water absorption capacity (189 g/g) without using any cross-linker, concluding that a shorter curing time (half an hour) prevents the formation of a tighter hydrogel network induced by heat.

Das et al.^50^ formulated a hydrogel using carboxymethylcellulose and hydroxyethylcellulose cross-linked with citric acid. Hydrogels cross-linked with 1% citric acid exhibited weak bonding, resulting in lower water absorbency (<2 g/g). On the other hand, formulations containing 3% citric acid showed a reduced absorption capacity due to the increased density of cross-linking bonds, which restricted water permeability through the polymeric network. Optimal results were obtained with 2% citric acid, resulting in approximately 6 g/g of water absorption. This trend was also observed in a study by Fu et al.^34^

Huang et al.^61^ employed epichlorohydrin as a cross-linker, leading to a remarkable increase in water absorption capacity from 180 to 1220 g/g (Figure 4). Qi et al.,^62^ using epichlorohydrin as a cross-linker in a CMC and β-cyclodextrin hydrogel, reported a mass swelling capacity of 8725 g/g after 3 h of immersion. This highlights that epichlorohydrin has the potential to serve as an effective cross-linker when combined with cellulose.

For starch, seven different cross-linkers were reported: citric acid (5 events), MBA (9 events), and no cross-linkers (3 events), as well as trimetaphosphate (TTP), CaCl_2_, glutaraldehyde, and epichlorohydrin (each with 1 event) (Figure 5). The forest plots highlighted variations in water absorption capacity for starch when used with different cross-linkers, with the order of effectiveness being MBA > no cross-linker > citric acid [ORs 3.55, 1.55, and 1.07, respectively].

**Figure 5:**
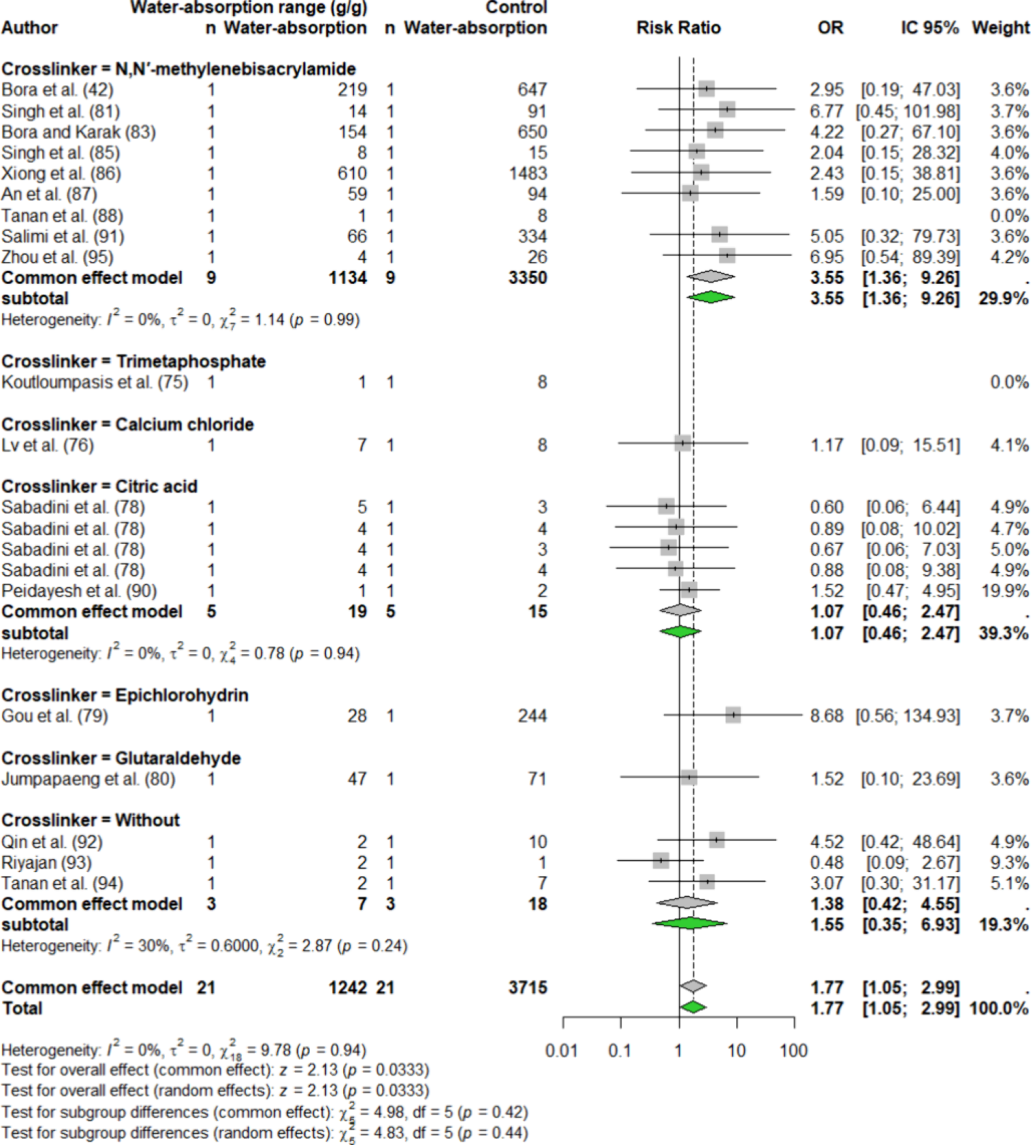
Forest plot representing starch-based hydrogels, detailing maximum and minimum water absorption capacities (g/g), odds ratios (ORs), summary effects, 95% confidence intervals (CIs), and the percentage of weight attributed to each study. The analysis follows an REM, with a 95% CI for the mean effect size under fixed-effect meta-analysis.

The MBA cross-linker exhibited a range of water absorption capacities, from a minimum of 1 to a maximum of 1483 g/g (Figure 4). However, the overall effect of MBA on the starch hydrogels was not statistically significant (*p* > 0.05), indicating that the cross-linker had no substantial influence on the water absorption capacity of starch-based hydrogels. Xiong et al.^86^ demonstrated that the water absorption capacity of the material increased from 610 to 1483 g/g with 0.08% w/w MBA.

The use of CaCl_2_ as a cross-linking agent appeared in five studies, though these were not included in the meta-analysis, as they involved alginate-based hydrogels. Among divalent metal cations, calcium salts are commonly used as cross-linkers for alginate due to their low cost and nontoxicity. Alginate, particularly in the form of homopolymeric G-blocks, has a strong affinity for Ca^2+^ ions. When alginate is exposed to a CaCl_2_-containing medium, the carboxyl groups in the polymer bind to the Ca^2+^ ions, leading to the rapid cross-linking of alginate chains in aqueous solutions. This process forms the characteristic “egg-box” structure through the stacking of G-blocks (Figure S5).^114,123^

The impact of CaCl_2_ concentration on hydrogel properties is demonstrated in the study by Arafa et al.,^82^ where different concentrations of CaCl_2_ were used to cross-link an alginate and chitosan hydrogel. The mass swelling capacity achieved values of 4.37, 3.83, and 3.73 g/g for CaCl_2_ concentrations of 1%, 3%, and 5% (w/w), respectively. As the concentration of CaCl_2_ increases, the hydrogel network becomes denser, leading to a reduced flexibility of the polymer chains and consequently limiting water diffusion through the matrix.

In chemical cross-linking, the degree of cross-linking will influence, among other properties, the structural network of the hydrogel and consequently its mass swelling capacity. The selection of a cross-linking agent depends on the specific components of the hydrogel as each cross-linker requires particular conditions, such as pH and temperature, for optimal reaction efficiency. However, some chemical cross-linkers can pose a risk to human health and the environment, necessitating careful evaluation for safe application.

Synthetic cross-linkers such as *N*,*N*′-methylenebis­(acrylamide), epichlorohydrin, and glutaraldehyde are frequently utilized to enhance the structural integrity and absorption capacity of hydrogels. However, these compounds are classified as potentially harmful, and their exposure to environmental stressors such as drought and rewetting cycles, temperature, and pH conditions has an impact on the efficiency and integrity of materials. Over time, hydrogels synthesized with them can degrade into persistent, ecotoxic fragments that deposit on the soil, presenting substantial drawbacks to the environment. Given these concerns, the development of hydrogels using natural cross-linking agents, such as citric acid, rather than synthetic compounds, is often preferred as a strategic shift toward more sustainable practices.^103,129,130^

### Further Agricultural Applications

Table 5 highlights various hydrogel-loading materials, such as nutrients, chemical compounds, and microorganisms. In addition to serving as water reservoirs, hydrogels can act as carriers for these materials, potentially enhancing agriculture.^10^

**Table 5:** Articles on Hydrogels Loaded with Different Compounds

principal components	load	reference
cellulose and gelatin	NPK fertilizer	^31^
cellulose	urea	^32^
cellulose, kaolin, and diatomite	urea	^34^
cellulose	urea	^39^
cellulose and starch	NPK fertilizer	^42^
cellulose and keratina	copper(II) chloride	^49^
cellulose and acid whey	urea, potassium nitrate	^52^
cellulose and polyvinyl alcohol	NPK fertilizer	^54^
cellulose and alginate	urea	^58^
cellulose and β-cyclodextrin	urea	^62^
cellulose	urea	^66^
starch	potassium chloride, ammonium chloride	^74^
starch and alginate	urea	^76^
starch, natural rubber, and montmorillonite	urea	^80^
starch, gelatin, and agar	thiophanate methyl	^81^
starch and alginate	thiophanate-methyl	^85^
starch, natural rubber, and polyvinyl alcohol	urea	^88^
starch and natural char	urea	^91^
starch and montmorillonite	urea	^95^
chitosan and benzaldehyde	urea	^99^
chitosan, PVA, and montmorillonite	NPK	^100^
chitosan and K-carrageenan	urea	^101^
alginate	urea, NPK fertilizer	^120^
alginate	potassium nitrate, dipotassium phosphate,	^123^
alginate and natural soil colloid	acetamiprid	^125^
cellulose and alginate	tomato and potato leaf extract	^131^
cellulose, biochar, and montmorillonite	triple superphosphate fertilizer	^132^
carrageenan	urea	^133^
starch and chitosan	,	^134^
starch and gelatin	potassium nitrate	^135^
chitosan and montmorillonite	NPK fertilizer	^136^
pectin and natural rubber	golden banana extract	^137^
gelatin and xanthan gum	urea	^138^
collagen	nitrogen, potassium	^139^
cashew gum	dipotassium phosphate	^140^
kaolin and polyvinyl alcohol	diammonium hydrogen phosphate	^141^
gum tragacanth	urea, calcium nitrate	^142^

Fertilizers can be either coated onto or integrated within the hydrogel matrix. The feasibility of this depends on the material’s properties (i.e., thickness, porosity, and solubility), which may require blending with other components to ensure effective fertilizer retention. Slow-release fertilizers have been developed to control the gradual release of nutrients, prolonging their bioavailability in the soil throughout crop growth cycles. This controlled release minimizes nutrient loss through leaching and reduces the risk of root damage, thus improving the overall efficacy of the fertilizer compared to conventional methods of application.^30,143^

For hydrogels to effectively retain water and control the release of essential agricultural components, it is critical to optimize their composition to enhance their efficiency in the field.^144,145^ For example, Dou et al.^136^ demonstrated that incorporating NPK fertilizer into a chitosan and MMT hydrogel reduced nutrient release on the first day from 81.5% in a pure chitosan hydrogel to 74.6% and 68.4% with the addition of 3.3% and 10% w/w MMT, respectively. The denser network formed by increasing the MMT content helped regulate nutrient release more effectively. Similarly, Bora et al.^42^ incorporated NPK into a hydrogel made from starch and wastepaper powder. On the first day, nutrient release did not exceed 15%, but by the twentieth day, N, P, and K release reached 98%, 81%, and 95%, respectively.

E et al.^32^ utilized a CMC and cellulose nanofiber hydrogel for controlled release of urea, observing less than 20% nitrogen (N) release by day 5, and about 90% by day 30. Similarly, Li et al.^133^ evaluated a carrageenan-based hydrogel under various environmental conditions. In water, over 50% of nitrogen was released on the first day, with a gradual decline leading to more than 90% release by day 28. In soil, the same hydrogel showed a slower release rate, with 16.44% and 16.83% N release on the first day at 25 and 35 °C, respectively, and about 75% by the twenty-eighth day.

Incorporating bioactive compounds into hydrogels offers a green alternative for plant protection against pathogens.^146^ For instance, Clemente et al.^131^ added tomato leaf extracts to CMC beads and tested their biocidal activity. Among the bacteria and fungi evaluated, the highest inhibition zones were observed against and , with halos of 22 and 20 mm, respectively. Singh et al.^85^ incorporated thiophanate-methyl into a hydrogel made of starch, alginate, and polyacrylamide, noting that an increase in MBA content led to more cross-linking points in the network. This reduced the swelling capacity and slowed the diffusion of the fungicide, prolonging its release over 72 h. Similarly, Zhang et al.^125^ incorporated acetamiprid into an alginate-based hydrogel with 7.5% w/w soil colloid, observing a 34.4% release of acetamiprid over 54 h, a slower release compared to hydrogels made with alginate alone.

In addition to chemical compounds, hydrogels can also be used to incorporate plant growth-promoting bacteria. Perez et al.^134^ immobilized Az39 and ZME4 cells, either separately or coimmobilized, in chitosan and starch hydrogel networks. The results showed that within the first 6 days, immobilized ZME4 and Az39 released 10^7^ and 10^5^ CFU/mL, respectively. Furthermore, coimmobilized bacteria were released more rapidly than those immobilized individually.

Joshi et al.^123^ encapsulated in alginate beads and tested its effects on larvae. The study showed that, compared to the powdered form of , the encapsulation in alginate beads slowed the mortality rate, with 70% of the larvae population killed over 144 h. This delayed effect was attributed to the controlled release of from the bead network, which prolonged its action over time.

Hydrogels also function as water reservoirs, enhancing the soil moisture content critical for plant growth. Additionally, the network structure of the absorbents enables improved application by incorporation of fertilizers, agrochemicals, and microorganisms within their matrix. These incorporated compounds become more available to plants compared to direct soil application, as they are gradually released from the hydrogel matrix via water diffusion.^10^

### Future Trends

Hydrogels are 3D polymeric networks with diverse applications across various fields, including agriculture. In agriculture, hydrogels act as water reservoirs, providing essential moisture to promote plant growth. Additionally, they serve as carriers for the controlled release of a variety of beneficial components into agricultural crops. Based on current trends and emerging innovations, future prospects point to a landscape of opportunities and challenges, including:

1. Utilizing agricultural and industrial waste as a source of natural polymers to reduce the cost of hydrogel production;
2. Chemically modifying natural polymer structures to enhance hydrogel performance;
3. Modifying the three-dimensional structure by combining different polymers and natural cross-linking agents to enable multiple water absorption/release cycles, making hydrogels suitable for perennial and long-term crops;
4. Developing hydrogel formulations with environmentally friendly components to ensure that no undesirable or harmful substances remain in the soil postharvest, facilitating the replacement of synthetic absorbents;
5. Assessing the water dynamics of hydrogels in varying soil types (i.e., textures and compositions) is essential for a clearer understanding of hydrogel behavior and enhancing the effectiveness of hydrogels under diverse agricultural conditions;
6. Biopolymer-based hydrogels, though biodegradable, may require frequent reapplication due to degradation. As such, their ecological impacts and effectiveness over time, particularly compared to synthetic alternatives, need to be carefully assessed;
7. Evaluating the effectiveness of hydrogels as carriers for different plant defense and plant growth-promoting microorganisms, aiming to increase agricultural productivity;
8. Advancing the industrial scalability of hydrogel production processes.

Therefore, biobased hydrogels can mitigate the effects of climate change on agricultural crops by providing protection from extended periods of drought and preventing soil compaction, which in turn may facilitate optimal root development. Furthermore, the tunability of the absorbent material enables diversification of their properties, thereby meeting the diverse hydrogel usage requirements.

## Conclusions

Water absorption capacity is one of the most critical properties of hydrogels for agricultural applications. The studies included in this Perspective demonstrate a clear correlation between the natural polymers used in hydrogel production and their water absorption capacity. Hydrogels composed of biopolymer mixtures and cross-linking agents generally exhibit higher water absorption capacity than those made from a single biopolymer. Therefore, it is essential to carefully assess the proportions of the materials used in hydrogel production to optimize their performance.

The choice of components in the hydrogel matrix determines the type and concentration of cross-linking agents, which, in turn, directly influences the final properties of the hydrogel. Cellulose-based hydrogels, for instance, show significant swelling capacity, particularly when cross-linked with *N*,*N*′-methylenebis­(acrylamide). However, it is important to note that many of the cross-linkers used in the studies reviewed may pose environmental risks, necessitating careful evaluation to ensure safe application.

The potential for further improvement of the hydrogels is substantial. The incorporation of chemical compounds and microorganisms can promote soil and plant health. However, research on the immobilization of microorganisms in the hydrogel matrix remains limited, which presents an important area for future investigation.

## Supplementary Material



## Data Availability

Data supporting this study are included within the manuscript.

## Notes

Table with water absorption of biobased hydrogels; representation of structures of cellulose, starch, and alginate; funnel plot for bias analysis; forest plot representing biobased hydrogels separated by biopolymers (PDF)
