# Supplementary material for: Water Absorption Capacity and Agricultural Utility of Biopolymer-Based Hydrogels: A Systematic Review and Meta-Analysis
Source: ACS Polym Au. 2025 Jun 11;5(4):325–42. doi: 10.1021/acspolymersau.5c00019 (PMC12355620; doi:10.1021/acspolymersau.5c00019)
Supplement: Supplementary file 1 [file lg5c00019_si_001.pdf]

Supporting Information

Water absorption capacity and agricultural utility of biopolymer-based hydrogels: A systematic review and meta-analysis

Guilherme Schwingel Henn[1,2], Caroline Schmitz[1], Liliana Berté Fontana[2], Heloisa Vieceli Nunes Corrêa[1], Daniel Neutzling Lehn[1,2], Claucia Fernanda Volken de Souza[1,2,*]

[1] Laboratory of Food Biotechnology, University of Vale do Taquari, Lajeado, RS, Brazil.

[2] Graduate Program in Biotechnology, University of Vale do Taquari, Lajeado, RS, Brazil.

* Corresponding author: Av. Avelino Tallini, 171, ZC 95914-014, Lajeado, RS, Brazil. E-mail: claucia@univates.br

Table S1. Summary of studies on bio-based hydrogels, including adjuvant materials, cross-linking agents, and results for the lowest and highest water absorption capacities (WAC) with respective immersion times in water.

Biomaterials	Monomers	Cross-linker	Lowest WAC (g/g)	Highest WAC (g/g)	Immersion time (h)	References
Agar and gum Arabic	Acrylic acid	MBA	1.34	3.78	-	(1)
Agarose	Activated carbon	-	9.2*	14.5*	24	(2)
Amla	Methacrylic acid	MBA	1.03*	5.14*	24	(3)
Carboxy-methyl tamarind	Methacrylic acid	MBA	54.94*	248.88*	24	(4)

kernel gum						
Carrageenan	Polyvinyl alcohol and polyvinylpyrrolidone	Glutaraldehyde	-	4.58*	24	(5)
Carrageenan and palm frond	Polyvinyl alcohol and polyvinylpyrrolidone	Glutaraldehyde	4.45*	5.52*	24	
Carrageenan	Acrylic acid	MBA	43.72	514.04	24	(6)
Carrageenan, montmorillon, and psyllium	-	-	23.14	28.93	12	(7)
Cashew gum	Acrylamide	MBA	-	1100	-	(8)
Cashew gum	Acrylamide	MBA	150*	240*	2	(9)
Collagen	Acrylic acid and maleic anhydride	MBA	611*	2208	-	(10)
Fibroin	-	-	2.3*	91	12	(11)
Fly ash	Sodium polyacrylate	MBA	255*	310	6	(12)
Gelatin and xanthan gum	-	-	11.81*	25.00	24	(13)
Gelatin, lignin, and hemicellulose	Acrylic acid and acrylamide	MBA	39	825	24	(14)
Gelatin and agar-agar	Acrylic acid and methyl acrylate	MBA	1.30*	6.36*	18	(15)
Gellan gum	-	Citric acid	40	77	24	
Gellan gum and bentonite	-	Citric acid	31	62	24	(16)
Gellan gum and halloysite	-	Citric acid	39	67	24	
Gellan gum	Acrylic acid	MBA	2.53	5.56	-	(17)

Guar gum	Methyl methacrylate	Polylactic acid	-	25.74*	10	(18)
Guar gum and zeolite	-	PEG-600	10.12	92.50	0.5	(19)
Guar gum	Acrylic acid	MBA	104*	189*	24	(20)
Guar gum	Graphene oxide	Borax	-	1.22*	24	(21)
Guar gum	-	Borax	-	1.52*	24	
Guar gum	Acrylic acid and acrylamide	MBA	133.8*	168.2*	2.5	(22)
Guar gum	Acrylic acid and NIPAM	MBA	158.7*	220.9*	2.5	
Guar gum	Acrylic acid	EGDMA	98.69	809.20	12	(23)
Gum tragacanth	Acrylamide and methacrylic acid	Glutaraldehyde	1.64	2.59	-	(24)
Kaolin	Polyvinyl alcohol	-	28.40*	94.01*	4	(25)
Kaolin	Acrylic acid and acrylamide	-	117.80	159.60	4	(26)
Keratin	Acrylic acid and acrylamide	MBA	1.51	9.90	24	(27)
Lignin and agarose	-	Epichlorohydrin	4.59*	17.99*	2	(28)
Lignin	Polyvinyl alcohol	Epichlorohydrin	39	570	72	(29)
Lignosulfonic acid sodium salt and γ-Polyglutamic acid	-	Polyglycol diglycerol	60.34	364.12	24	(30)
Lignosulfonic acid sodium salt	Acrylic acid	MBA	39.68	560.24	-	(31)
Locust bean	-	-	47.4*	127.4*	0.67	(32)

gum and borax						
Oil shale semicoke	Acrylic acid and acrylamide	MBA	171.50 *	643.13	4	(33)
Okra mucilage	Acrylic acid	EGDMA	-	1.11*	24	(34)
Pectin and natural rubber	Acrylic acid	MBA	5.32	14.10	-	(35)
Porcine plasma protein	Glycerol	Glutaraldehyde	1.94*	36.91	24	(36)
Salecan	Acrylamide and itaconic acid	MBA	17.80*	61.01*	6	(37)
Soy Protein Isolate	Glycerol	-	1.85*	20.40	24	(38)
Soy Protein Isolate	Glycerol	-	12.61*	36.44	24	(39)
Watermelon rind	Acrylic acid and acrylamide	MBA	242*	744*	24	(40)
Xanthan gum, wood fibers, and glycerol	-	-	-	7.60*	24	
Xanthan gum, wood fibers, and glycerol	-	Citric acid	2.65*	4.19*	24	
Xanthan gum, wood fibers, and glycerol	-	Tannic acid	2.62*	5.61*	24	(41)
Xanthan gum, wood fibers, and glycerol	-	Sodium trimetaphosphate	3.00*	4.51*	24	

WAC: Water absorption capacity. Values with a (*) represent data collected with ImageJ. EGDMA: ethylene glycol dimethacrylate; MBA: N, N′-methylenebisacrylamide; NIPAM: N-isopropyl acrylamide; PEG-600: polyethylene glycol.

References

(1) Hasija, V.; Sharma, K.; Kumar, V.; Sharma, S.; Sharma, V. Green synthesis of agar/Gum Arabic based superabsorbent as an alternative for irrigation in agriculture. *Vacuum* **2018**, 157, 458–464.

(2) Cao, L.; Li, N. Activated-carbon-filled agarose hydrogel as a natural medium for seed germination and seedling growth. *Int. J. Biol. Macromol.* **2021**, 177, 383–391.

(3) Farooq, K.; Kumar, V.; Sharma, V.; Bhagat, M.; Kumar, V.; Sharma, K. Synthesis, optimization, and multifunctional evaluation of amla-based novel biodegradable hydrogel. *Polym. Bull.* **2024**, 81, 10681–10705.

(4) Malik, R.; Warkar, S. G.; Saxena, R. Carboxy-methyl tamarind kernel gum based bio-hydrogel for sustainable agronomy. *Mater. Today Commun.* **2023**, 35, 105473.

(5) Elessawy, N. A.; Alhamzani, A. G.; Almahmoud, S. A. J.; Hsiao, B. S. Evaluation, optimization study, and life cycle assessment of novel eco-friendly PVA-based nanocomposite hydrogel adsorbents for methylene blue and paracetamol removal. *Ecotoxicol. Environ. Saf.* **2024**, 285, 117123.

(6) Li, J.; Zhu, Y.; Liu, M.; Liu, Z.; Zhou, T.; Liu, Y.; Cheng, D. Network interpenetrating slow-release nitrogen fertilizer based on carrageenan and urea: A new low-cost water and fertilizer regulation carrier. *Int. J. Biol. Macromol.* **2023**, 242, 124858.

(7) Aydınoğlu, D.; Karaca, N.; Ceylan, Ö. Natural Carrageenan/Psyllium Composite Hydrogels Embedded Montmorillonite and Investigation of Their Use in Agricultural Water Management. *J. Polym. Environ.* **2021**, 29, 785–798.

(8) Barros, D. M. A.; Edvan, R. L.; Pessoa, J. P. M.; do Nascimento, R. R.; Camboim, L. F. R.; Bezerra, L. R.; de Araújo, M. J.; de Sousa, H. R.; Silva-Filho, E. C. Hydrogel Based on Cashew Gum and Polyacrylamide as a Potential Water Supplier in Mombaça Grass Pastures: A Sustainable Alternative for Agriculture. *Sustainability* **2023**, 15, 16423.

(9) Rodrigues Sousa, H.; Lima, I. S.; Neris, L. M. L.; Silva, A. S.; Santos Nascimento, A. M. S.; Araújo, F. P.; Ratke, R. F.; Silva, D. A.; Osajima, J. A.; Bezerra, L. R.; Silva-Filho, E. C. Superabsorbent Hydrogels Based to Polyacrylamide/Cashew Tree Gum for the Controlled Release of Water and Plant Nutrients. *Molecules* **2021**, 26, 2680.

(10) Hu, Z. -Y.; Chen, G.; Yi, S. -H.; Wang, Y.; Liu, Q.; Wang, R. Multifunctional porous hydrogel with nutrient controlled-release and excellent biodegradation. *J. Environ. Chem. Eng.* **2021**, 9, 106146.

(11) Cheng, K.; Tao, X.; Qi, Z.; Yin, Z.; Kundu, S. C.; Lu, S. Highly Absorbent Silk Fibroin Protein Xerogel. *ACS Biomater. Sci. Eng.* **2021**, 7, 3594–3607.

(12) Rattan, B.; Dhobale, K. V.; Saha, A.; Garg, A.; Sahoo, L.; Sreedeep, S. Influence of inorganic and organic fertilizers on the performance of water-absorbing polymer amended

soils from the perspective of sustainable water use efficiency. *Soil Till. Res.* **2022**, 223, 105449.

(13) Das, S.; Dalei, G. In situ forming dialdehyde xanthan gum-gelatin Schiff-base hydrogels as potent controlled release fertilizers. *Sci. Total Environ.* **2023**, 875, 162660.

(14) Kenawy, E. -R.; Seggiani, M.; Hosny, A.; Rashad, M.; Cinelli, P.; Saad-Allah, K. M.; El-Sharnouby, M.; Shendy, S.; Azaam, M. M. Superabsorbent composites based on rice husk for agricultural applications: Swelling behavior, biodegradability in soil and drought alleviation. *J. Saudi Chem. Soc.* **2021**, 25, 101254.

(15) Chaudhary, J.; Thakur, S.; Sharma, M.; Gupta, V. K.; Thakur, V. K. Development of Biodegradable Agar-Agar/Gelatin-Based Superabsorbent Hydrogel as an Efficient Moisture-Retaining Agent. *Biomolecules* **2020**, 10, 939.

(16) Sabadini, R. C.; Fernandes, M.; Bermudez, V. de Z.; Pawlicka, A.; Silva, M. M. Hydrogels Based on Natural Polymers Loaded with Bentonite and/or Halloysite: Composition Impact on Spectroscopic, Thermal, and Swelling Properties. *Molecules* **2024**, 29, 131.

(17) Choudhary, S., Sharma, K., Bhatti, M. S., Sharma, V., Kumar, V. DOE-based synthesis of gellan gum-acrylic acid-based biodegradable hydrogels: screening of significant process variables and in situ field studies. *RSC Adv.* **2022**, 12, 4780–4794.

(18) Paswan, M.; Prajapati, V.; Dholakiya, B. Z. Optimization of biodegradable cross-linked guar-gum-PLA superabsorbent hydrogel formation employing response surface methodology. *Int. J. Biol. Macromol.* **2022**, 223, 652–662.

(19) Songara, J. C.; Patel, J. N.; Mungray, A. A. Preparation and characterization of PAA/ GG-zeolite nano-composite hydrogel for agricultural applications. *J. Indian Chem. Soc.* **2022**, 99, 100686.

(20) HaqAsif, A.; Karnakar, R. R.; Sreeharsha, N.; Gite, V. V.; Borane, N.; Al-Dhubiab, B. E.; Kaliyadan, F.; Rasool, T.; Nanjappa, S. H.; Meravanige, G. pH and Salt Responsive Hydrogel based on Guar Gum as a Renewable Material for Delivery of Curcumin: A Natural Anti-Cancer Drug. *J. Polym. Environ.* **2021**, 29, 1978–1989.

(21) E, J. S. C.; Gopi, S.; A, R.; G, S.; Pius, A. Highly crosslinked 3-D hydrogels based on graphene oxide for enhanced remediation of multi contaminant wastewater. *J. Water Process Eng.* **2019**, 31, 100850.

(22) Abdel-Raouf, M. E.; El-Saeed, S. M.; Zaki, E. G.; Al-Sabagh, A.M. Green chemistry approach for preparation of hydrogels for agriculture applications through modification of natural polymers and investigating their swelling properties. *Egypt. J. Pet.* **2018**, 27, 1345–1355.

(23) Thombare, N.; Mishra, S.; Siddiqui, M. Z.; Jha, U.; Singh, D.; Mahajan, G. R. Design and development of guar gum based novel, superabsorbent and moisture retaining hydrogels for agricultural applications. *Carbohydr. Polym.* **2018**, 185, 169–178.

(24) Saruchi; Kumar, V.; Mittal, H.; Alhassan, S. M. Biodegradable hydrogels of tragacanth gum polysaccharide to improve water retention capacity of soil and environment-friendly controlled release of agrochemicals. *Int. J. Biol. Macromol.* **2019**, 132, 1252–1261.

(25) Sharma, N.; Singh, A.; Dutta, R. K. Biodegradable fertilizer nanocomposite hydrogel based on poly(vinyl alcohol)/kaolin/diammonium hydrogen phosphate (DAhP) for controlled release of phosphate. *Polym. Bull.* **2021**, 78, 2933–2950.

(26) He, R.; Tan, Y.; Chen, H.; Wang, Z.; Zhang, J.; Fang, J. Preparation and properties of novel superabsorbent polymer (SAP) composites for cementitious materials based on modified metakaolin. *Constr. Build. Mater.* **2020**, 258, 119575.

(27) Arican, F.; Uzuner-Demir, A.; Sancakli, A.; Ismar, E. Synthesis and characterization of superabsorbent hydrogels from waste bovine hair via keratin hydrolysate graft with acrylic acid (AA) and acrylamide (AAm). *Chem. Pap.* **2021**, 75, 6601–6610.

(28) de Albuquerque, T. L.; Cavalcante, V. G. C.; da Silva Rocha, W.; de Macedo, A. C.; Rocha, M. V. P. Hydrogels based on lignin extracted from cashew apple bagasse and its application in antimicrobial wound dressings. *Int. J. Biol. Macromol.* **2024**, 262, 130169.

(29) Wu, L.; Huang, S.; Zheng, J.; Qiu, Z.; Lin, X.; Qin, Y. Synthesis and characterization of biomass lignin-based PVA super-absorbent hydrogel. *Int. J. Biol. Macromol.* **2019**, 140, 538–545.

(30) Ma, Q.; Huang, W.; Xu, W.; Zhou, H.; Hashan, D.; She, D. Synergistic mechanisms of lignin-based novel materials and leaf-surface selenium fertilizer in alleviating drought and heavy metal stress. *Ind. Crops Prod.* **2024**, 222, 119487.

(31) Nandal, M.; Gupta, R. K.; Kumar, D. Synthesis, characterization and application of Lignosulphonate-g- poly(sodium acrylate) hydrogel. *Indian J. Chem. Technol.* **2023**, 30, 753–764.

(32) Chen, X.; Yang, T.; Cai, X.; Liu, Y.; Huang, C.; He, J.; Tian, D.; Yang, G.; Shen, F.; Zhang, Y. Eco-friendly hydrogel based on locust bean gum for water retaining in sandy soil. *Int. J. Biol. Macromol.* **2024**, 275, 133490.

(33) Wang, Y.; Zhu, Y.; Mu, B.; Liu, Y.; Wang, A. From the Waste Semicoke to Superabsorbent Composite: Synthesis, Characterization and Performance Evaluation. *J. Polym. Environ.* **2021**, 29, 4017–4026.

(34) Choudhary, V.; Sharma, S.; Shukla, P.Kr.; Malik, A. Biocompatible stimuli responsive hydrogels of okra mucilage with acrylic acid for controlled release phytochemicals of *Calendula officinalis*: *In vitro* assay. Mater. Today. **2023**, DOI: 10.1016/j.matpr.2023.03.701.

(35) Abdul Sattar, O. D.; Khalid, R. M.; Yusoff, S. F. M. Eco-friendly natural rubber-based hydrogel loaded with nano-fertilizer as soil conditioner and improved plant growth. *Int. J. Biol. Macromol.* **2024**, 280, 135555.

(36) Álvarez-Castillo, E.; Pelagio, M. J.; Bengoechea, C.; Guerrero, A. Plasma based superabsorbent materials modulated through chemical cross-linking. *J. Environ. Chem. Eng.* **2021**, 9, 105017.

(37) Qi, X.; Wei, W.; Su, T.; Zhang, J.; Dong, W. Fabrication of a new polysaccharide-based adsorbent for water purification. *Carbohydr. Polym.* **2018**, 195, 368–377.

(38) Álvarez-Castillo, E.; Del Toro, A.; Aguilar, J. M.; Guerrero, A.; Bengoechea, C. Optimization of a thermal process for the production of superabsorbent materials based on a soy protein isolate. *Ind. Crops Prod.* **2018**, 125, 573–581.

(39) Cuadri, A. A.; Romero, A.; Bengoechea, C.; Guerrero, A. The Effect of Carboxyl Group Content on Water Uptake Capacity and Tensile Properties of Functionalized Soy Protein-Based Superabsorbent Plastics. *J. Polym. Environ.* **2018**, 26, 2934–2944.

(40) Teng, B.; Zhong, Y.; Wu, J.; Zhu, J.; Cai, L.; Qi, P.; Luo, Z. Transforming watermelon (*Citrullus lanatus*) rind into durable superabsorbent hydrogels for enhanced soil water retention properties and adsorbs dye in water. *Heliyon* **2024**, 10, e38656. https://doi.org/10.1016/j.heliyon.2024.e38656

(41) Sorze, A.; Valentini, F.; Burin Mucignat, M.; Pegoretti, A.; Dorigato, A. Multifunctional xanthan gum/wood fibers based hydrogels as novel topsoil covers for forestry and agricultural applications. *Carbohydr. Polym. Technol. Appl.* **2024**, 7, 100520.

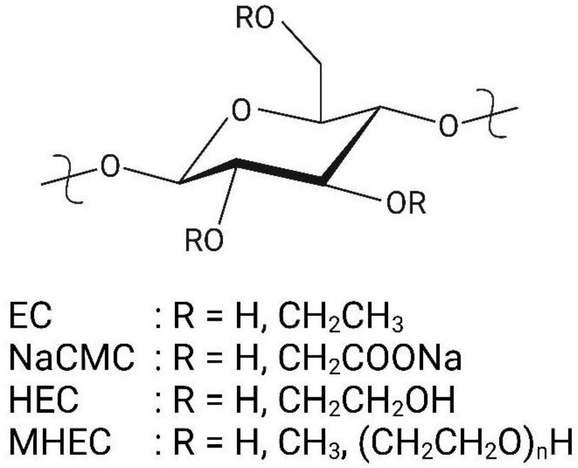

Figure S1. General structure of cellulose and common functional substituents used to derive cellulose-based polymers. EC: Ethylcellulose; NaCMC: Sodium carboxymethylcellulose; HEC: Hydroxyethylcellulose; MHEC: Methyl 2-hydroxyethyl cellulose. This figure was created using BioRender (https://www.biorender.com/), under a Creative Commons license.

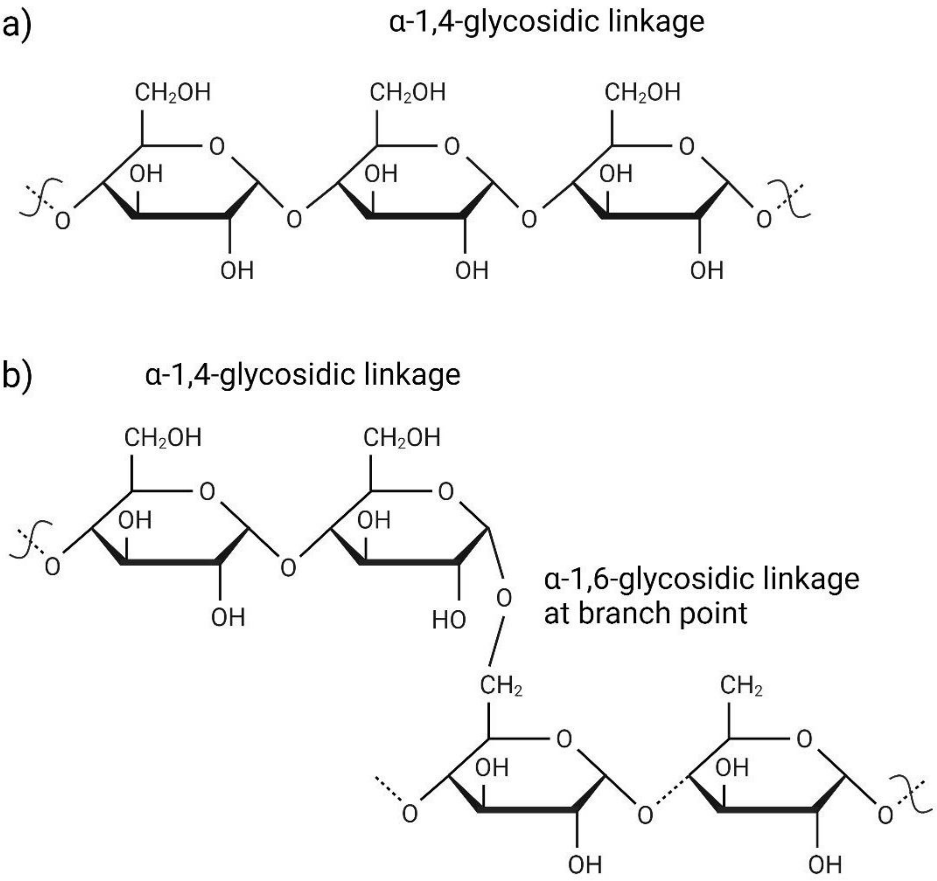

Figure S2. Representation of α-1,4 and α-1,6 glycosidic bonds in starch, forming linear (a) and branched chains (b). This figure was created using BioRender (https://www.biorender.com/), under a Creative Commons license.

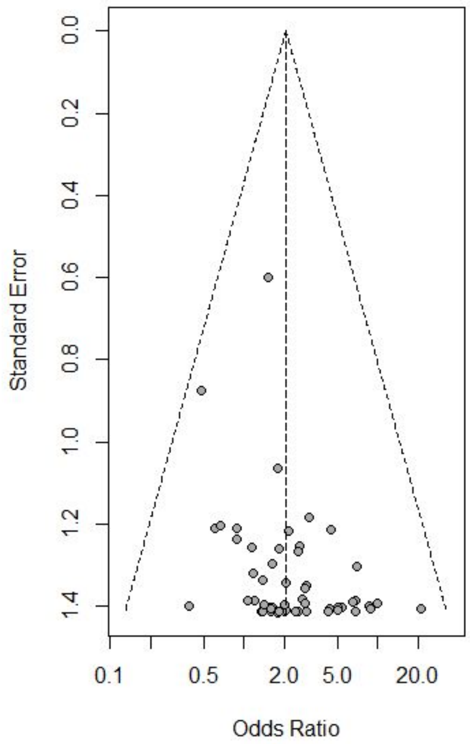

Figure S3. Funnel plot illustrating potential publication bias among the 64 studies included in the meta-analysis.

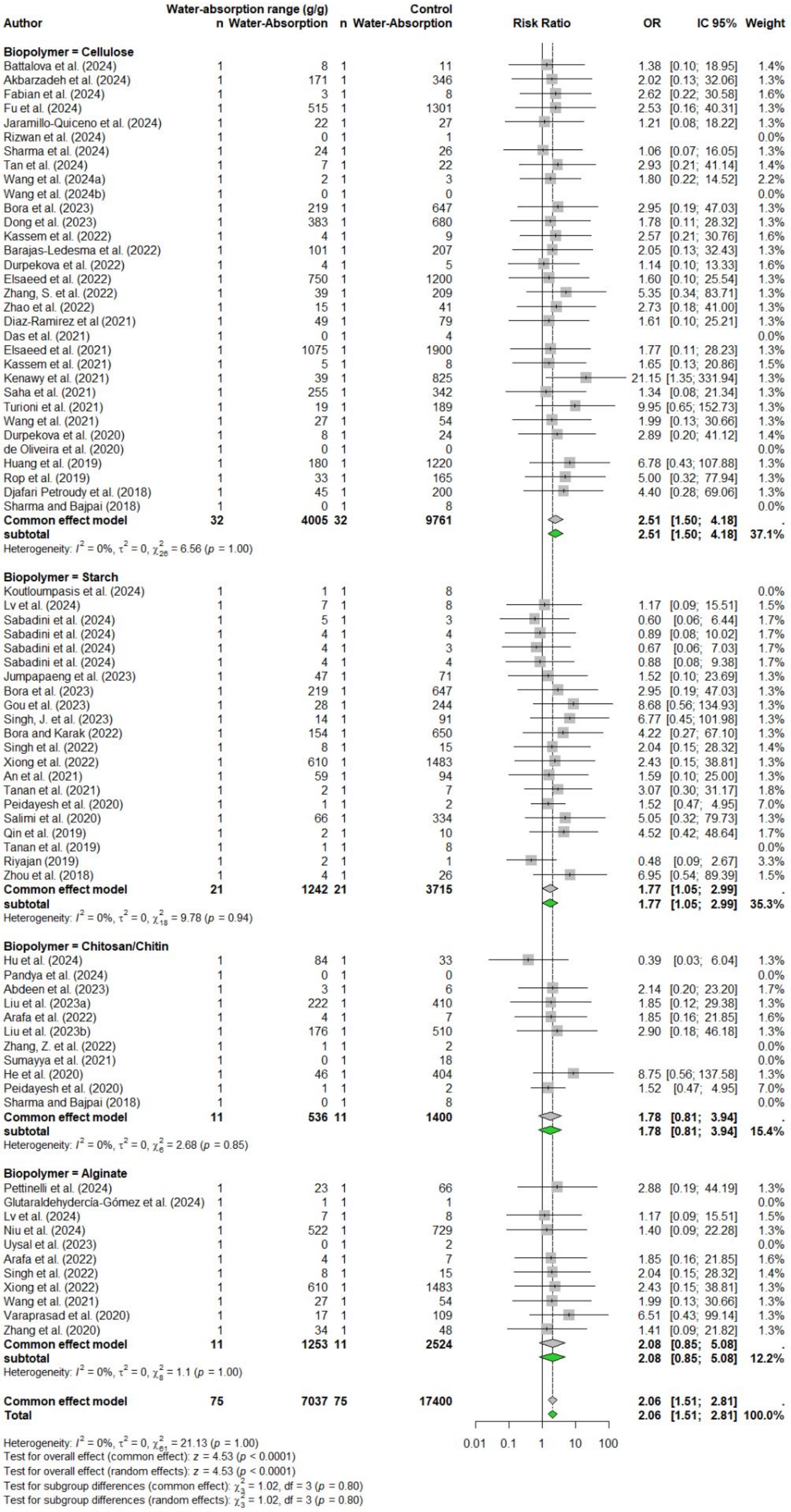

Figure S4. Forest plot representing bio-based hydrogels, detailing maximum and minimum water absorption (g/g), odds ratios (OR), summary effect, 95% confidence intervals (CI), and the

percentage of weight attributed to each study. The analysis follows a random-effects model, with a 95% CI for the mean effect size under fixed-effect meta-analysis.

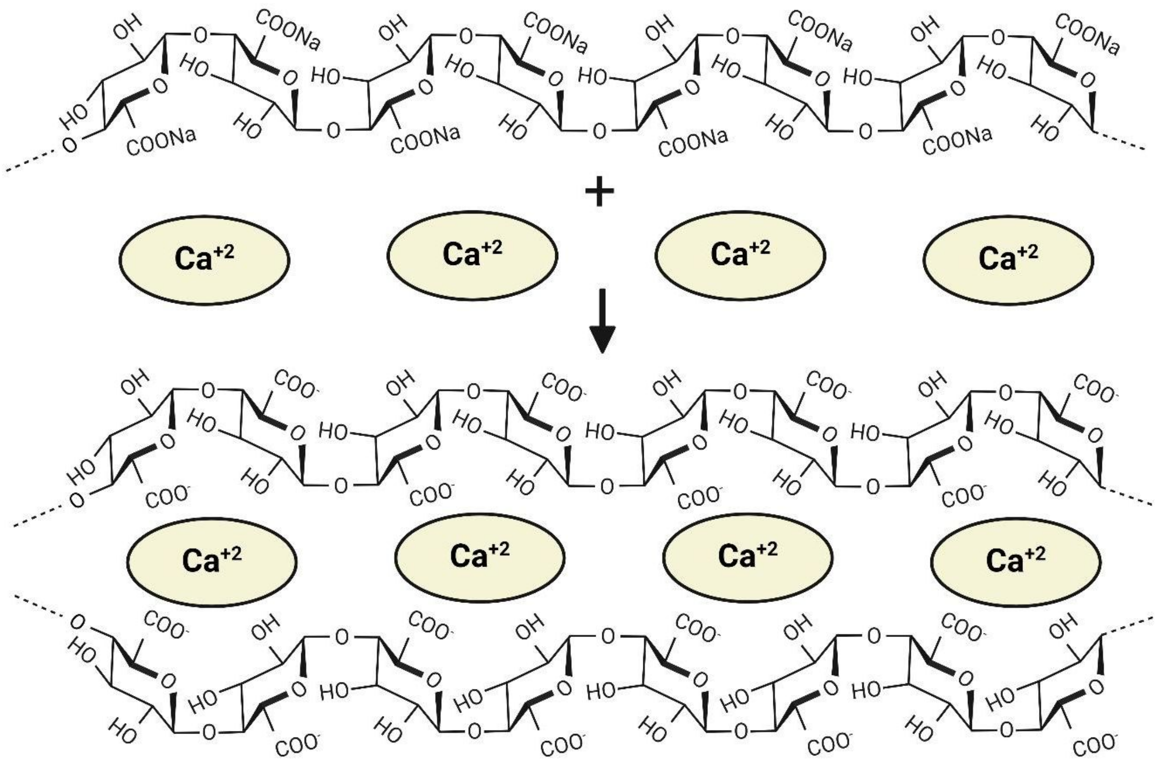

Figure S5. Schematic representation of the "egg-box" structure formed by reticulation of sodium alginate chains in the presence of calcium ions (Ca^{2+}). This figure was created using BioRender (https://www.biorender.com/), under a Creative Commons license.
